# Microplasma-synthesized *Citrus*-derived carbon quantum dots: antibacterial properties and nanoprobe sensitivity

**DOI:** 10.1039/d5ra02150b

**Published:** 2025-07-17

**Authors:** Nguyen Minh Hoa, Le Duc Toan, Ngo Tran, Le Xuan Hung, Le Anh Thi

**Affiliations:** a Faculty of Fundamental Sciences, Hue University of Medicine and Pharmacy, Hue University Hue 530000 Vietnam; b Natural Sciences Department, Phu Yen University Tuy Hoa City Phu Yen 5600 Vietnam; c Institute of Research and Development, Duy Tan University Da Nang 550000 Vietnam leanhthi@duytan.edu.vn; d Faculty of Natural Sciences, Duy Tan University Da Nang 550000 Vietnam

## Abstract

This study introduces an environmentally friendly and cost-efficient approach for producing carbon quantum dots (CQDs) from *Citrus nobilis deliciosa via* a microplasma-assisted technique. The obtained CQDs demonstrated excitation-dependent fluorescence accompanied by a red shift, which can be ascribed to quantum size effects and the influence of surface chemical functionalities. The synthesized CQDs demonstrated remarkable antibacterial properties, achieving growth inhibition rates of 99.24% against *Staphylococcus aureus* and 98.12% against *Escherichia coli* at a concentration of 50 μg mL^−1^. The antibacterial mechanism was primarily driven by membrane destabilization and oxidative stress induction, making CQDs a promising alternative to conventional antimicrobial agents. Additionally, the CQDs served as highly responsive fluorescent probes for Cd(ii) ion detection, exhibiting a linear response range spanning 1–14 μg mL^−1^, a minimum detectable concentration of 0.12 μg mL^−1^, and a Stern–Volmer quenching constant (*K*_SV_) of 0.45 μg mL^−1^. These findings highlight the dual functionality of CQDs as potent antibacterial agents and efficient fluorescence-based sensors for heavy metal detection. The eco-friendly synthesis, combined with the excellent biocompatibility and adjustable optical characteristics of CQDs, highlights their potential for applications in biosensing, environmental monitoring, and biomedical fields.

## Abstract 2

*Citrus* waste was converted into carbon quantum dots (CQDs) *via* one-step microplasma synthesis. The CQDs show antibacterial activity and act as fluorescent probes, offering green dual-functionality for biomedical applications.

## Graphical abstract

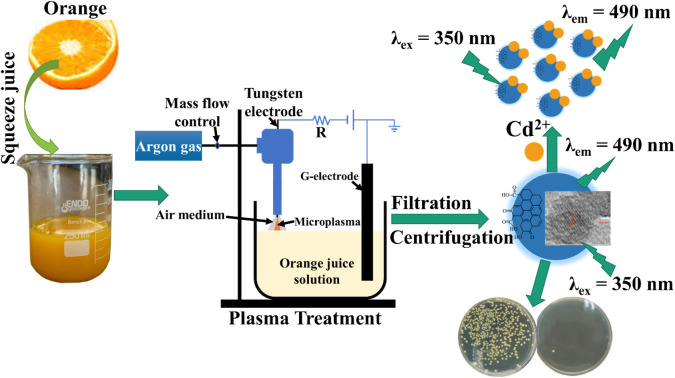

## 1 Introduction

Carbon quantum dots (CQDs) constitute an emerging category of fluorescent, metal-free nanoparticles (NPs) distinguished by a carbonaceous core encased within amorphous carbon layers. Owing to their remarkable attributes including minimal toxicity, excellent photostability, superior water solubility, outstanding biocompatibility, and a simple, cost-efficient synthesis process CQDs have attracted widespread research interest.^1,2^ CQDs can be synthesized through two primary strategies: decomposition-based (top-down) and construction-based (bottom-up) approaches. In the top-down route, larger carbon-based materials are disintegrated into nanoscale dots using techniques such as laser fragmentation, electrochemical methods, or oxidative processes. Conversely, the bottom-up approach involves building CQDs from molecular precursors through methods like hydrothermal or solvothermal treatments, microwave-assisted synthesis, and plasma-based processes.^3,4^ CQDs possess tunable optical properties, attributable to their nanoscale dimensions and the abundance of surface functional moieties such as hydroxyl (–OH) and carboxyl (–COOH) groups, which enhance their excellent aqueous solubility and colloidal stability.^5^ These nanoparticles contain sp^2^ or sp^3^-hybridized carbon frameworks that endow them with strong photoluminescent behavior, thereby enabling their use in a wide range of applications, including optoelectronic devices, chemical biological sensing, photocatalysis, bioimaging, biosensor development, antimicrobial treatments,^6^ fingerprint detection.^7^

Carbon-based nanomaterials, particularly CQDs, demonstrate superior performance compared to other quantum dots (QDs), such as II–VI semiconductors QDs.^8^ CQDs are notably biocompatible, making them safer for biomedical applications and environmentally friendly by eliminating the toxicity concerns associated with heavy metals in traditional QDs.^9,10^ They also exhibit excellent photostability and tunable optical properties that rival or even surpass those of conventional QDs. The ease of preparation, economic viability, and multifunctionality of CQDs across diverse fields from analytical sensing to energy storage systems further contribute to their widespread interest and utility.^7,8^ In summary, carbon-based nanomaterials, especially CQDs, offer unparalleled advantages for a wide array of applications.

As previously mentioned, various methods for preparing CQDs have been developed. However, many of these methods involve toxic precursors, stringent reaction conditions, complex separation processes, and often result in low quantum yields. For example, Jiang *et al.* synthesized carbon dots using phenylenediamine, a highly toxic precursor, and required prolonged reaction times.^11^ Similarly, Wu *et al.* investigated the formation of N-doped graphene nanodots using dicyandiamide and citric acid, followed by a 3-hour reaction and 48 hours of dialysis to remove unreacted small molecules and solvent.^12^ Furthermore, many CQDs produced through these methods exhibit low PL quantum yields.^13,14^ The synthesis of GQDs at high temperatures and over extended reaction times also often results in the production of chemical by-products and uncontrollable reaction processes, leading to non-uniform sizes and compromised luminescence quantum yields. There is a significant need for the development of optimal synthesis methods that are both economically viable and environmentally friendly to improve the quality of CQDs.

Recently, CQDs have been produced using the green hydrothermal method with precursors derived from carbon-rich natural sources such as banana, lemon juice, aqueous leaf extracts of tulsi,^15^ henna leaf,^16^ mango leaf extracts,^17^ and mung bean seeds.^18^ These CQDs typically exhibit quasi-spherical grain sizes ranging from 5–10 nm, are well-dispersed, and possess high photostability, although their quantum yield is below 10%. These characteristics make them suitable for applications in heavy metal detection and antibacterial treatments. Microplasma technology is emerging as a promising method for synthesizing nanomaterials,^19^ particularly due to its ability to control NPs sizes through plasma–liquid interaction treatment.^20^ This method, conducted low temperature and atmospheric pressure, is advantageous for achieving uniform size distribution. The plasma method has been predominantly used for synthesizing metal NPs such as gold (Au), copper (Cu), and silver (Ag) with sizes from 20 to 150 nm. These metal NPs have demonstrated significant antibacterial effectiveness by penetrating bacterial cells and addressing bacterial resistance.^21,22^ However, their applications in clinical settings poses challenges due to potential toxicity and destructive mechanisms, leading to complex interactions with cells. Thus, ongoing research is focused on developing NPs that can mitigate these issues while maintaining their.

For the fabrication of carbon NPs, precursors such as fructose, citric acid, ammonium citrate, and folic acid have been utilized.^23^ These precursors provide active radicals, including H, O, and OH, which facilitate the surface functionalization of carbon NPs without the need for additional solvents.^14^ Coupling carbon NPs with biomolecules or natural compounds helps control their size and ensures they are biodegradable, biocompatible, and non-toxic.^24^ CQDs are an emerging group of luminescent nanoparticles, typically composed of a carbonaceous core enclosed within disordered or amorphous carbon structures. The antibacterial efficacy and non-toxic nature of CQDs are closely linked to their size and surface charge properties. These properties can be modified *via* surface functionalization, which involves the introduction of specific chemical groups such as amides, amino, hydroxyl, carboxyl, and epoxy groups. This controlled adjustment allows for fine-tuning the antibacterial characteristics and biocompatibility of CQDs, making them suitable for various applications in medical and biotechnological fields.^25^ The bactericidal activity of CQDs primarily involves membrane destruction and the generation of reactive oxygen species.^26^ Recent studies have demonstrated that quantum dots with precisely engineered surface functionalities can exert potent antibacterial effects through mechanisms involving reactive oxygen species (ROS) generation, membrane destabilization, and electrostatic interactions with bacterial cell walls. These mechanisms contribute to cell wall rupture, oxidative stress, and ultimately bacterial cell death, making such nanomaterials promising candidates for antimicrobial applications.^27^ Furthermore, the positively charged radical functionalities present on the CQD surfaces are capable of electrostatic interaction with the negatively charged bacterial membranes of species like *Escherichia coli* (*E. coli*) and *Staphylococcus aureus* (*A. aureus*).^28^ Therefore, CQDs are a promising class of materials that can be developed for use as antibacterial agents.

CQDs have important applications in water treatment and metal ion sensing, especially in aquatic environments.^29^ Detecting Cd^2+^ ions in water is particularly crucial, as contamination from industrial sources or corroded piping can compromise drinking water quality. Elevated concentrations of Cd^2+^ not only alter the taste of drinking water but may also lead to severe health complications, including nausea, gastrointestinal disturbances, abdominal pain, and hepatic toxicity when exposure exceeds permissible thresholds.^30^ While CQDs are commonly used as fluorescent probes to detect Cd^2+^, their effectiveness is often limited by poor stability and low fluorescence quantum yield. These drawbacks restrict their broader use in areas like information encryption and biological imaging.

In this study, we synthesized the CQDs using *Citrus nobilis deliciosa* (orange juice) as a green carbon source, free from acid or alkaline reagents, employing the microplasma method at atmospheric pressure and low temperature. The tunable fluorescence response of CQDs under varying excitation wavelengths renders them particularly advantageous for utilization in biomedical applications. The formation of CQDs involves the carbonization of the major components in *Citrus nobilis deliciosa*, such as citric acid, sucrose, fructose, glucose, and ascorbic acid. We also explored the antibacterial applications of these CQDs, demonstrating their efficacy against *S. aureus* and *E. coli* species and metal ion sensing.

Despite notable advancements in CQD research, many existing synthesis techniques still rely on toxic precursors, high-temperature processes, or elaborate post-treatment steps. These limitations hinder the scalability, cost-effectiveness and biocompatibility required for real-world biomedical and environmental applications. While several recent “green” approaches have explored the use of plant extracts, fruit peels, or food waste as carbon sources, such strategies often result in low photoluminescence quantum yield, inconsistent particle size, or limited control over surface functionalization, which restrict their broader utility. Microplasma-assisted synthesis has recently emerged as a powerful alternative for producing nanomaterials under ambient pressure and low-temperature conditions. This method provides several, advantages, including precise control over particle size, *in situ* surface functionalization, and elimination of hazardous chemical reagents. However, its application for synthesizing CQDs from natural, renewable carbon sources remains relatively underexplored in the current literature.

In this work, we present a sustainable, reagent-free microplasma-assisted synthesis of CQDs using *Citrus nobilis deliciosa* as a low-cost and eco-friendly carbon precursor. The resulting CQDs exhibit dual functional properties: (1) excellent antibacterial activity against both *E. coli* and *S. aureus*, and (2) highly sensitive and selective fluorescence-based detection of cadmium (Cd^2+^) ions. The novelty of this study lies in the integration of a green synthesis pathway with multifunctional nanomaterials performance, thus offering a scalable and biocompatible platform for applications in biosensing, environmental monitoring, and antimicrobial therapy.

## 2 Experimental

### 2.1 Materials

Fresh *Citrus nobilis deliciosa* fruits were sourced from a nearby market. All experimental procedures utilized double-distilled water, which was prepared using an Aquatron A4000D purification unit. Analytical-grade metal salts such as Na^+^, Cu^2+^, Mg^2+^, Ca^2+^, and Zn^2+^, which were sourced from Sigma-Aldrich.

### 2.2 Carbon dot synthesis

**Scheme 1:**
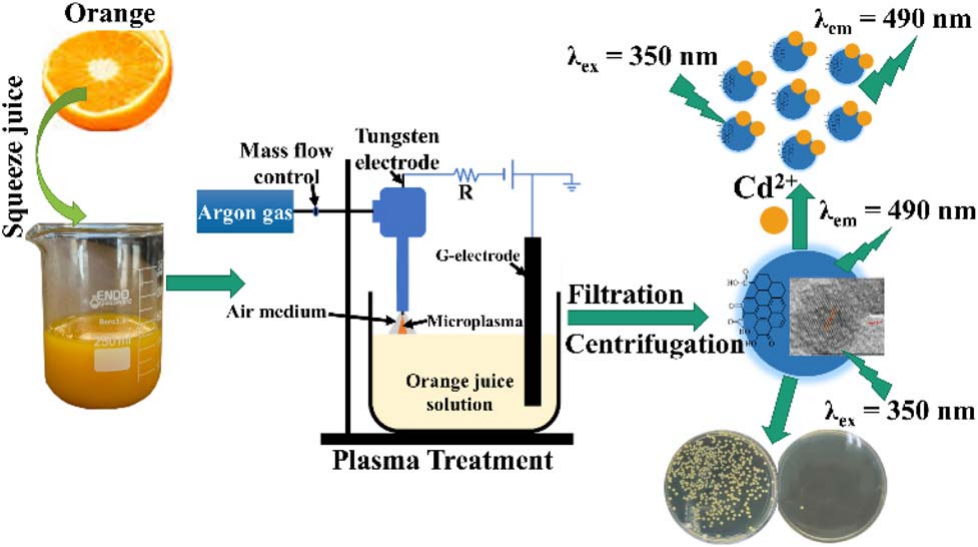
Schematic diagram of the micro-plasma system for the synthesis of carbon quantum dots.

The microplasma synthesis of CQDs was conducted using a plasma system as illustrated in Scheme 1 and described in a previous study.^31^ An aliquot of 20 mL *Citrus nobilis deliciosa* extract was transferred into a clean glass beaker. The CQDs were synthesized using a microplasma-assisted technique with *Citrus nobilis deliciosa* juice as the carbon source. The microplasma system consisted of a stainless-steel needle-type anode and a platinum wire cathode, both immersed directly into 20 mL of freshly extracted *Citrus* solution contained in a glass beaker. A direct current (DC) high-voltage power supply, adjustable up to 2 kV, was employed to initiate and maintain the plasma discharge. The discharge was operated at a constant current of 5 mA, with the applied voltage stabilized around 1.6 kV during the process. The plasma was generated under atmospheric pressure in ambient air, without the use of any external carrier or reactive gas, ensuring an environmentally benign and reagent-free synthesis environment. The plasma treatment was carried out for 15 minutes, during which the solution temperature of the solution was carefully monitored and maintained below 60 °C to avoid thermal degradation of organic components. This setup enabled efficient plasma–liquid interaction, facilitating carbonization and surface functionalization of the resulting CQDs in a single step. Subsequently, the mixture was passed through filter paper to eliminate residual solids or impurities. Ethanol was then added to the aqueous filtrate, and the mixture was centrifuged at 12 000 rpm for 15 minutes to separate the precipitate. The resultant product was redispersed in water for fluorescence characterization.

### 2.3 Characterizations of CQDs

The absorption properties of the as-prepared CQDs were characterized using ultraviolet-visible (UV-Vis) spectroscopy (V-570, Jasco). Photoluminescence (PL) properties were investigated using fluorescence spectroscopy (Fluorog 4, Horiba Jobin-Yvon). The morphology, size distribution, and structural uniformity of the particles were examined using transmission electron microscopy (TEM) and high-resolution TEM (HRTEM) performed on a JEOL 2100 instrument. The structural characteristics of the CQDs were examined using micro-Raman spectroscopy (XploRA PLUS, Horiba Jobin Yvon) with a 532 nm excitation laser. Surface functional groups were characterized by Fourier transform infrared spectroscopy (FTIR) utilizing a Bruker Alpha T instrument. The X-ray photoelectron spectroscopy (XPS) was performed using a NEXSA (Thermo Fisher Scientific). All spectroscopic analyses were conducted under ambient conditions.

### 2.4 Antibacterial examination

The antibacterial characteristics of the CQDs were evaluated using the growth kinetics approach. Cultures of *E. coli* and *S. aureus* were grown in nutrient broth and maintained at 37 °C for a period of 24 to 48 hours under incubation. Subsequently, both bacterial strains were propagated in Mueller–Hinton broth, and suspensions were prepared using 0.9% sterile saline to reach a turbidity equivalent to 0.5 McFarland standard, corresponding to approximately 10^8^ CFU mL^−1^ (colony-forming units). A 50 μL aliquot of the CQD solution was then dispensed onto agar plates previously seeded with *E. coli* and *S. aureus* cultures. The plates were incubated at 37 °C for 24 hours. The inhibition zones around the CQD drops were observed visually and compared to those produced by gentamicin to evaluated the antibacterial. Antimicrobial efficacy was evaluated by enumerating the remaining colony-forming units (CFUs) after treatment. The percentage of bacterial inhibition was determined using the equation below:1
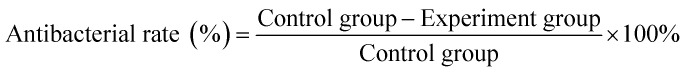


### 2.5 Detection of Cd^2+^

A fluorescence-based sensing system was developed to detect Cd^2+^ ions using the synthesized CQDs as nanoprobes. The detection assay was obtained by mixing 5000 μL of ultrapure water, 100 μL of CQDs solution (0.1 μg mL^−1^), and 100 μL of Cd^2+^ solution with varying concentrations ranging from 1 to 14 μg mL^−1^. The total volume was adjusted to 4 mL, ensuring a final CQD concentration of 2.5 μg mL^−1^. The mixture was allowed to react at room temperature for 1 minute before fluorescence spectra were recorded at an excitation wavelength of 350 nm. The fluorescence intensity of CQDs was systematically measured to determine the quenching effect induced by Cd^2+^ ions, enabling quantitative detection. A calibration plot was generated by correlating the ratio of fluorescence intensities (*I*_0_/*I*) with varying concentrations of Cd^2+^ ions, where *I*_0_ denotes the emission intensity without Cd^2+^ and *I* corresponds to the intensity observed in its presence. The results revealed a linear fluorescence quenching response over the tested concentration range, demonstrating the potential of CQDs as highly sensitive and selective Cd^2+^ sensors.

### 2.6 Interference studies

The selectivity of CQDs for Cd^2+^ detection was assessed in presence of potentially interfering metal ions. To evaluate the specificity of the CQD-based sensing system, fluorescence measurements were conducted in the absence and presence of 10 μg mL^−1^ of various competing metal ions, including Cu^2+^, Na^+^, Mg^2+^, Ca^2+^, and Zn^2+^, at room temperature.

## 3 Results and discussion

### 3.1 Structural characterization of CQDs

**Fig. 1:**
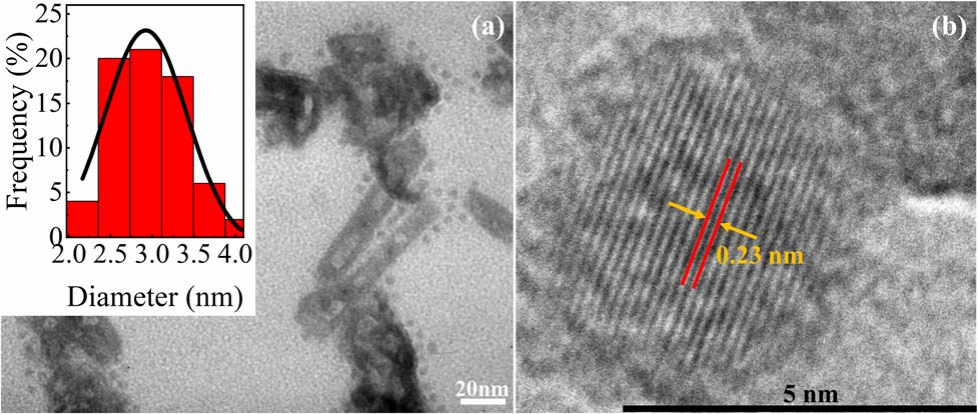
(a) TEM images of the CQDs are presented along with their corresponding particle size distribution histograms shown in the inset. (b) HRTEM image of CQDs.

The morphology and size distribution of CQDs were investigated using TEM and HRTEM, as depicted in Fig. 1. The TEM micrographs (Fig. 1(a)) reveal that the CQDs predominantly exhibit quasi-spherical morphology with relatively uniform size distribution. However, a minor fraction of elongated or rod-like nanostructures, as well as instances of particle aggregation, were also observed, likely due to local clustering during solvent evaporation. The particle size histogram, presented in the inset of Fig. 1(a) and fitted with a Gaussian distribution, indicated an average diameter of approximately 3.2 nm. This confirms the formation of ultrasmall nanoparticles with narrow size variation. Further structural analysis using HRTEM (Fig. 1(b)) demonstrates the crystalline nature of the CQDs, evidenced by a distinct interlayer lattice spacing of ∼0.23 nm, which corresponds to the (100) plane of graphitic carbon. This lattice spacing is in good agreement with previously reported CQDs synthesized *via* green and plasma-based methods,^32^ suggesting successful graphitization during plasma treatment.

**Fig. 2:**
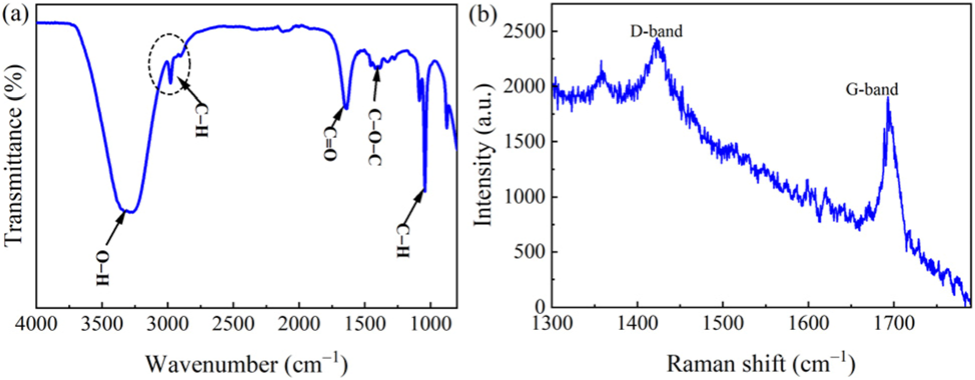
(a) FTIR, and (b) Raman spectra of CQDs.

The FTIR study was conducted to identify functional groups on the surfaces of the CQDs. As illustrated in Fig. 2(a), the FTIR spectrum of CQDs was recorded in a range of 400–4000 cm^−1^. The broad peak at 3330 cm^−1^ is assigned to the presence of the O–H bonds.^33^ The peak at 2953 cm^−1^ corresponds to C–H groups, confirming the existence of functionalized groups on the CQDs. This peak is observed in epoxy, carbonyl, and hydroxyl groups. The peaks at 1650 cm^−1^, 1389 cm^−1^, and 976 cm^−1^ can be attributed to the asymmetric and symmetrical stretches of the ether groups –C–O–C. The peaks in the 1241–1266 cm^−1^ and 1005–1135 cm^−1^ ranges are linked to C–H stretching.^33^ The appearance of –OH and C–O groups ensured excellent solubility of the CQDs in water and demonstrated their functionalization capability. The small size and high activity of CQDs enhance their potential as antibacterial agents. Furthermore, the structural characteristics of the CQDs were validated through Raman spectrometer analysis. As illustrated in Fig. 2(b), the Raman spectrum displays distinct peaks at 1453 and 1483 cm^−1^, which are assigned to the D and G bands characteristic of graphitic carbon structures, respectively. The D/G intensity ratio indicates a relatively high level of surface defects, which can play a role in the PL properties of the CQDs.

**Fig. 3:**
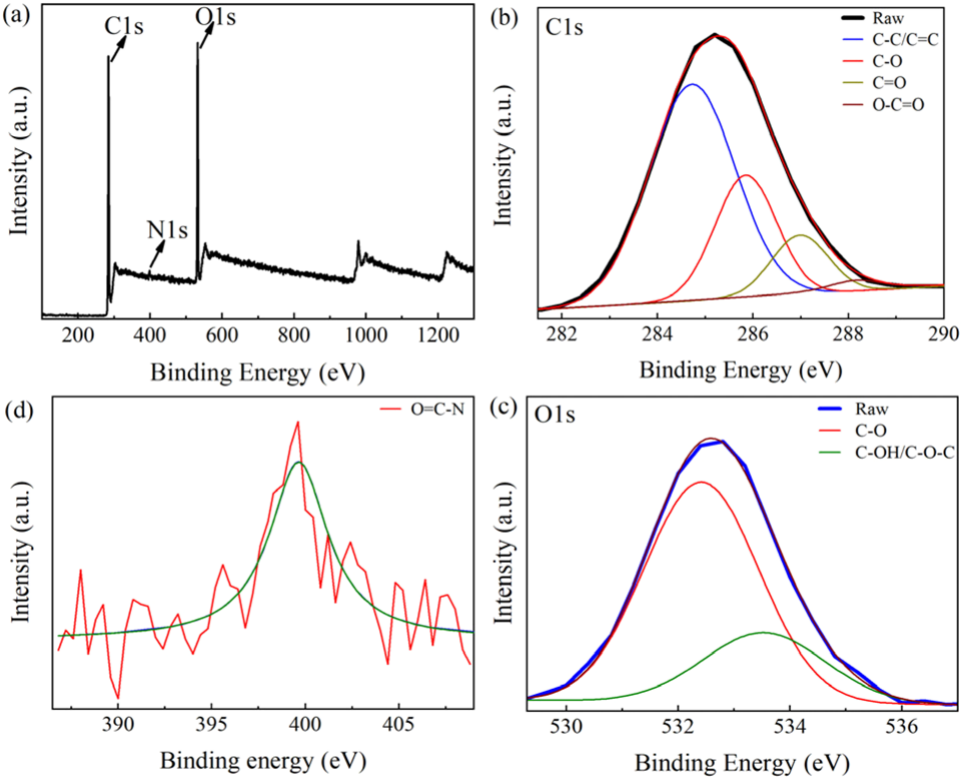
(a) XPS scan spectrum of CQDs and high-resolution XPS spectra of (b) C 1s, (c) O 1s, (d) N 1s.

The XPS was employed to investigate the elemental composition and surface chemistry of the synthesized CQDs, particularly to identify key surface functional groups.^25^ The wide-scan XPS spectrum (Fig. 3(a)) confirms the presence of three dominant elements: carbon (C 1s), oxygen (O 1s), and nitrogen (N 1s). Quantitative indicates that the atomic percentages are 78.99% C, 15.94% O, and 5.02% N, clearly indicating these are the primary constituents of the CQDs. Further insights into the chemical bonding environment are obtained from high-resolution C 1s spectrum (Fig. 3(b)), which were deconvoluted into four distinct peaks corresponding to: C–C/C=C at 284.7 eV (sp^2^-and sp^3^-hybridized carbon), C–O at 286.08 eV, C=O/C=N at 287.86 eV, and –COOH groups at 289.14 eV. The presence of the O=C–O peak is strong evidence for carboxylic acid functionalities on the CQD surface, supporting their role in Cd^2+^ coordination during sensing. This also indicates a relatively high degree of surface oxidation, which contributes to the CQDs' physicochemical and functional reactivity. The O 1s spectrum (Fig. 3(c)) further corroborates this, showing two peaks: 531.47 eV attributed to carbonyl (C=O) groups, and 532.95 eV corresponding to hydroxyl or ether (C–OH/C–O–C) groups.

Finally, the N 1s spectrum (Fig. 3(d)) displays a prominent peak at 399.13 eV, which is assigned to O=C–N bonding environments, suggesting that nitrogen-containing species likely derived from natural organic compounds in the *Citrus* precursor were successfully incorporated during plasma synthesis. These XPS findings confirm the presence of a variety of oxygen- and nitrogen-containing surface groups, which play critical roles in the optical, chemical, and biological functionality of the CQDs.

### 3.2 Optical properties of CQDs

**Fig. 4:**
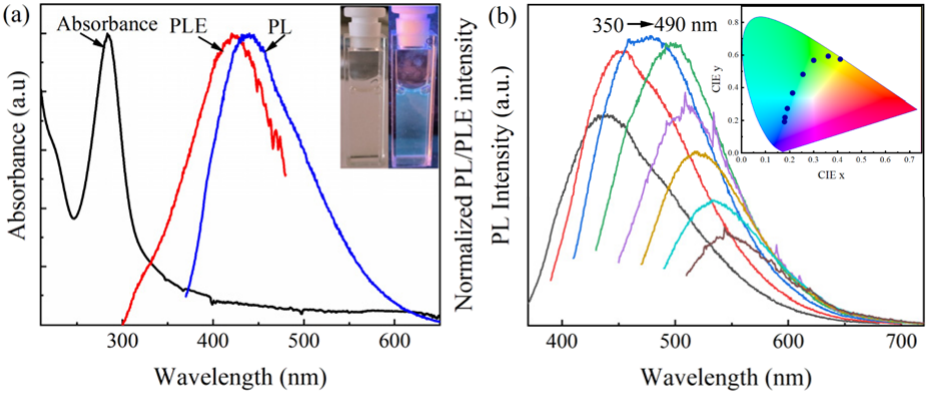
(a) UV-Vis absorption, emission, and excitation spectra of CQDs. Inset: a comparative analysis of the luminance emitted by CQDs under both natural daylight and a 254 nm light source, elucidating their versatile optical properties; (b) variation in the PL spectra with different excitation wavelengths of CQDs. Inset: visualization of the emitted light's color coordinates using the chromaticity diagram (CIE color mapping).

The optical properties of the CQDs were assessed through UV-Vis and fluorescence spectroscopy. Fig. 4(a) shows the UV-Vis spectrum of CQDs, with a prominent absorption peak at 284.3 nm and a into the UV zone, corresponding to π–π* transitions. These transitions are attributed to the C–O bond and the conjugated C–C bond a 254 nm light source, the CQDs exhibited blue luminescence as shown in the inset of Fig. 4(a). In the PL spectra, as shown in Fig. 4(b), the optimal emission and excitation wavelengths were 438 nm and 423 nm, respectively. The presence of the functional groups, specifically C atoms bonded with O atoms on the surface of the CQDs, creates emissive traps between π and π* states of C=C. Such interactions can influence the electronic configuration and bandgap of the carbon dots, thereby enabling their fluorescence under certain excitation wavelengths.^33^ Therefore, the PL mechanism of CQDs is influenced not only by their size but also by the presence of surface defects. The interplay between the dimensions of the CQDs and the characteristics of surface imperfections plays a significant role in shaping their PL behavior.

The fluorescence investigations of the CQDs revealed a discernible reliance of PL on the excitation PL across varying excitation wavelengths, as depicted in Fig. 4(b). Notably, as the excitation wavelengths spanned from 350 to 490 nm, a consequential displacement of the PL peaks from 438 to 549 nm was observed. This spectral shift is indicative of the nuanced modulation of CQD electronic structures under distinct excitation conditions This spectral shift is indicative of the nuanced modulation of CQD electronic structures under distinct excitation conditions. The CIE (chromaticity diagram) mapping in the inset of Fig. 4(b) visually substantiates this phenomenon, illustrating the chromatic shift in emitted light coordinates concomitant with excitation wavelength alterations. However, it is noteworthy that at higher excitation wavelengths, the intensity of PL exhibited a marked reduction. This diminution in intensity can be attributed to the interplay of CQD size and the presence of trap states.^34^ Such observations underscore the intricate interplay between CQD structural characteristics and optical properties, elucidating crucial insights into their photophysical behavior.

The analysis of CIE coordinates and their corresponding mapping delineated a noteworthy shift in the characteristic colors of emitted luminance, transitioning from blue to green in response to escalating excitation wavelength. This spectral evolution underscores the intricate interplay between the excitation conditions and the emissive characteristics of CQDs. The emergence of distinct emissive traps on the surface of CQDs likely contributes to this phenomenon. These trap states exhibit varying behavior in response to changes in excitation wavelength, thereby modulating the emission properties, as evidenced by alterations in the PL properties.^35^

### 3.3 Photostability study

**Fig. 5:**
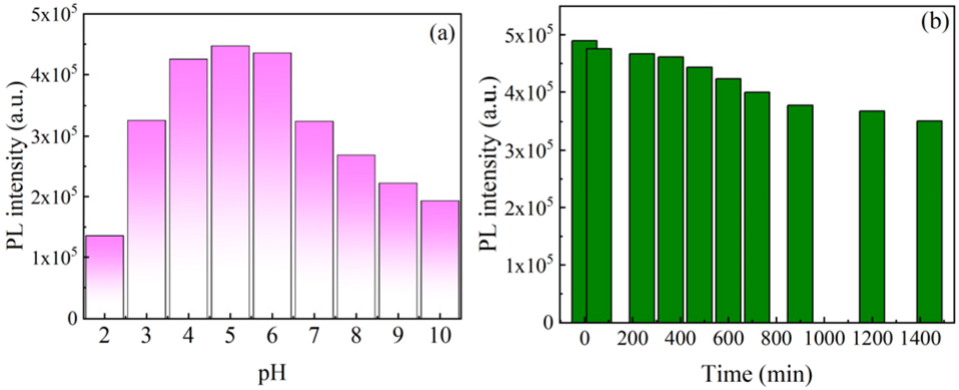
(a) PL stability of the CQDs at different pH values, (b) exposure to UV light (0–1440 min).

The photoluminescent stability, indicatives of a material's resistance to light and pH variations, is paramount for applications necessitating prolonged exposure. Fig. 5 presents the examined photoluminescent stability of the obtained CQDs. In the aqueous solution containing CQDs, stability was maintained without discernible particle degradation for over a month at room temperature. To assess the environmental responsiveness of the synthesized CQDs, their PL behavior was first investigated under varying pH conditions. As shown in Fig. 5(a), the emission intensity at 450 nm was strongly dependent on the solution's pH. The fluorescence was strongest in the mildly acidic range (pH 4–6), indicating that the surface states of the CQDs are highly sensitive to protonation and deprotonation. This pH-dependent PL behavior suggests the involvement of ionizable functional groups (*e.g.*, –OH, –COOH) on the CQD surface, which modulate the electronic environment and recombination pathways. To further investigate the optical robustness of the CQDs, we examined their photostability under continuous UV irradiation. As presented in Fig. 5(b), the emission intensity at 455 nm gradually declined during 24 hours of uninterrupted exposure to 245 nm UV light, with a total decrease of approximately 28.3%. This relatively slow rate of photobleaching indicates that the CQDs possess excellent resistance to photodegradation, maintaining a substantial proportion of their fluorescence over prolonged irradiation. Collectively, these results demonstrate that the synthesized CQDs exhibit both environmental sensitivity (*e.g.*, to pH) and strong optical stability, making them highly suitable for long-term fluorescence-based applications, such as biosensing, bioimaging, and environmental monitoring.

### 3.4 Antibacterial activity of the CQDs

**Fig. 6:**
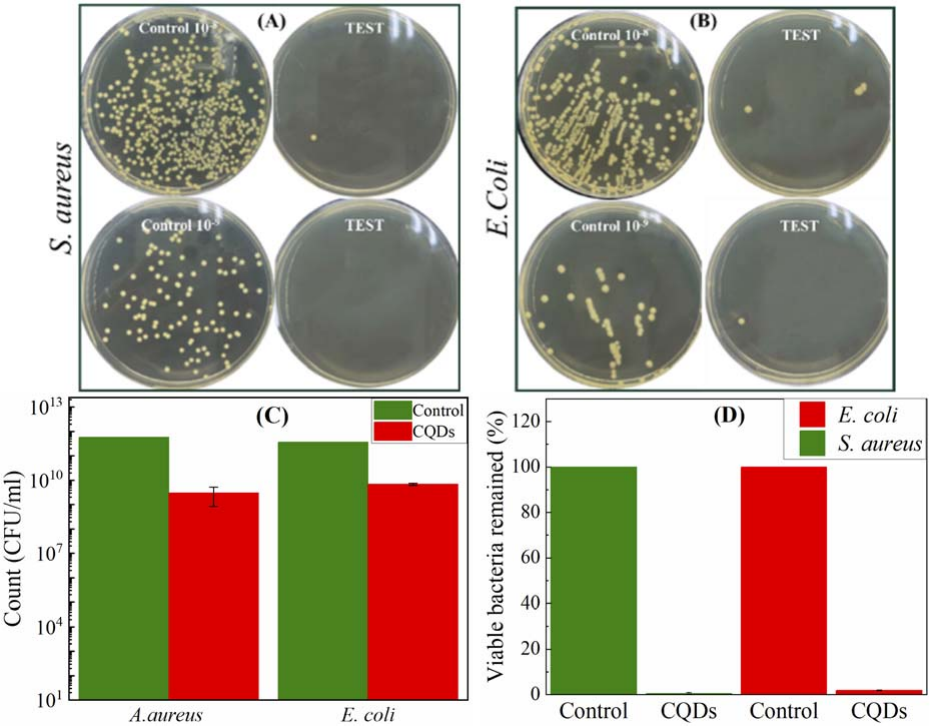
(A and B) Representative images and quantitative histograms illustrating colony counts of *S. aureus* and *E. coli* obtained from serial dilutions of homogenized infected tissue samples. (C and D) Corresponding statistical analysis following exposure to CQDs at a final concentration of 50 μg mL^−1^ for 25 hours under incubation at 37 °C.

The CQDs synthesized in this study represent a promising alternative to conventional antimicrobial nanomaterials, owing to their inherent surface charge, stable colloidal behavior, and potential for biocompatible ROS generation. To evaluate their antibacterial performance, we tested the CQDs against two representative bacterial strains: *Staphylococcus aureus* (Gram-positive) and *Escherichia coli* (Gram-negative). The experiments were conducted using three independent biological replicates, and the results as illustrated in Fig. 6(A–D). Bacterial viability was assessed using the standard plate count method, comparing untreated controls with CQD-treated cultures. As shown in Fig. 6C, significant reductions in colony-forming units (CFUs) were observed upon exposure to CQDs.

At a concentration of 50 μg mL^−1^, the CQDs achieved 98.12% growth inhibition for *E. coli* and 99.24% for *S. aureus* (Fig. 6D), indicating slightly higher sensitivity of the Gram-positive strain. The high antibacterial efficiency is attributed to multiple factors. First, the stable colloidal dispersion of the CQDs likely allows for sustained interaction with bacterial surfaces, enhancing their antimicrobial persistence. Second, the CQDs can induce oxidative stress through the generation of ROS. This mechanism can lead to cell membrane damage, protein dysfunction, and biofilm matrix disruption. These effects collectively contribute to reduced bacterial viability and enhanced inhibition zones.

A key contributor to the antibacterial mechanism is the electrostatic interaction between the positively charge CQDs and the negatively charged bacterial cell walls, which are rich in peptidoglycans containing –COOH and amino groups. This interaction can destabilize the bacterial membrane, cause cytoplasmic leakage, and ultimately result in cell lysis.^36^ To contextualize the efficacy of our CQDs, Table 1 summarizes antibacterial performance metrics of CQDs synthesized from various natural precursors using different methods. Notably, our microplasma-synthesized CQDs exhibit superior or comparable inhibition rates at lower concentrations relative to many previous reports. This highlights the advantages of our synthesis approach in producing highly active and sustainable nanomaterials for antibacterial applications. Taken together, the observed results support the conclusion that CQDs derived from *Citrus nobilis deliciosa* possess strong and broad-spectrum antibacterial properties, driven by both physicochemical surface interactions and ROS-mediated cytotoxicity. These findings reinforce the potential of CQDs as next-generation antimicrobial agents in biomedical and environmental applications.

**Table 1:** Comparison of CQD synthesized from different natural origins

Source of CQDs	Synthesis method	Target bacteria	Concentration	Inhibition rate	Ref.
*Citrus nobilis deliciosa*	Plasma	*S. aureus*	50 μg mL^−1^	99.24%, 98.12%	This work
		*E. coli*			
*Citrus* peel waste	Hydrothermal	*E. coli*	100 μg mL^−1^	95%, 97%	37
		*S. aureus*			
Green tea	Microwave	*S. aureus*	48.6 μg mL^−1^	21.3% to 79.3%	38
Banana peel	Pyrolysis	*E. coli*	150 μg mL^−1^	85%, 88%	39
		*B. subtilis*			
Waste Tea	Hydrothermal	*E. coli*	50 μg mL^−1^	81.6%	40
*Vitis Vinifera* Seeds	Hydrothermal	*S. aureus*	80 μg mL^−1^	>90%	41
		*S. mutans*			
		*E. coli*			
Palm oil	Hydrothermal	*E. coli*	10 μg mL^−1^	76%	42

### 3.5 CQDs ‘‘turn off’’ fluorescent probe for Cd(ii) detection

**Fig. 7:**
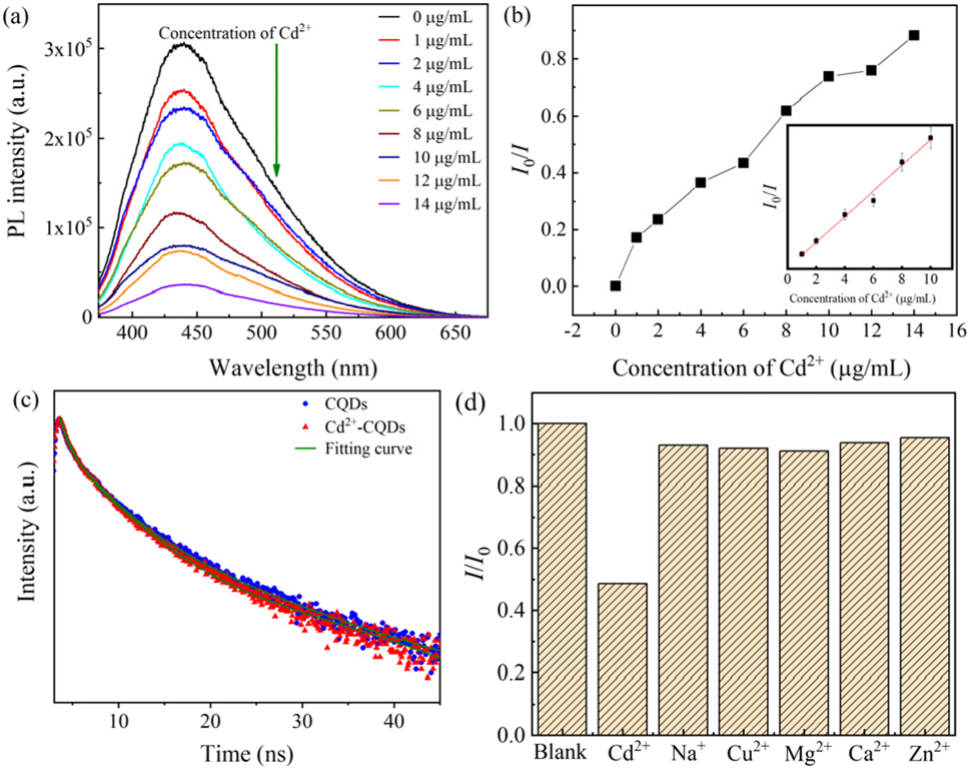
(a) Fluorescence spectra of different concentrations of Cd(ii) (from top to bottom: 0–14 μg mL^−1^) added to the CQDs solution at a fluorescence excitation wavelength of 350 nm; (b) dependence of fluorescence intensity on Cd(ii) concentration in the range of 0–14 μg mL^−1^ (the inset: linear relationship between fluorescence intensity and Cd(ii) concentration), (c) time-resolved decay curve of CQDs and CQDs prensent of Cd(ii), (d) selectivity of CQDs to Cd(ii) in the presence of other metal ions.

To evaluate the sensitivity of CQDs as a fluorescence probe for Cd^2+^ detection, the PL intensity was measured across Cd^2+^ a concentrations ranging from 0 to 14 μg mL^−1^ of Cd^2+^ ions. A progressive decrease in PL intensity was observed with increasing Cd^2+^ concentration, indicating a quenching effect (Fig. 7(a and b)). The fluorescence quenching observed upon addition of Cd^2+^ ions to the CQDs solution is attributed to surface-mediated interactions that interfere with the radiative recombination pathways of photoexcited electrons. This quenching behavior is closely linked to the chemical structure and surface functionalities of the CQDs, as characterized by FTIR and XPS analyses. Specifically, the CQDs surface is rich in hydroxyl (–OH) and carboxyl/carboxylate (–COOH/–COO^−^) groups, which act as electron-donating ligands or coordination sites for heavy metal ions. The Cd^2+^ ions, known for their high affinity toward oxygen-containing functional groups, can bind to these sites and form non-emissive coordination complexes at the CQD surface. This binding likely facilitates a photoinduced electron transfer (PET) process from the excited-state to the Cd^2+^ ions, resulting in fluorescence quenching.

The Stern–Volmer plot, shown in the inset of Fig. 7(b), displays a linear relationship at lower Cd^2+^ concentrations, consistent with either a dynamic (collisional) or static (complexation) quenching mechanism, depending on the specific interaction kinetics between Cd^2+^ and the surface-bound functional groups. Overall, the fluorescence sensing behavior of the CQDs is governed by their surface chemistry, particularly the presence of –COOH and –OH groups, which enable selective and sensitive recognition of Cd^2+^ ions through coordination-driven quenching pathways. These findings underscore the importance of surface functionalization in designing efficient CQD-based nanoprobes for heavy metal detection.

The correlation between fluorescence intensity (*I*_0_/*I*) and Cd^2+^ concentration follows the Stern–Volmer eqn (2):^43^

2 *I*_0_/*I* – 1 = 0.105 + 0.063[Cd^2+^]

In this expression, *I*_0_ and *I* correspond to the fluorescence intensities measured in the absence and presence of Cd^2+^ ions, respectively, while [Cd^2+^] denotes the concentration of cadmium ions in solution.

The CQDs synthesized from *Citrus nobilis deliciosa* using a plasma-assisted method exhibited a linear fluorescence response within the Cd^2+^ concentration range of 1–14 μg mL^−1^, with a correlation coefficient (*R*^2^) of 0.986, demonstrating high precision and reproducibility. The limit of detection (LOD) was determined to be 0.12 μg mL^−1^, calculated at a signal-to-noise ratio, highlighting the high sensitivity of the method. Additionally, the Stern–Volmer quenching constant (*K*_sv_) was 0.45 μg mL^−1^, confirming a strong affinity between the CQDs and Cd^2+^ ions. To contextualize the performance of the CQD sensor, a comparative analysis with other reported Cd^2+^ detection methods was conducted (Table 2). The comparison highlights variations in synthesis methods, detection techniques, linear range, and LOD among different Cd^2+^ detection methods.

**Table 2:** Comparison of other Cd^2+^ sensing performances using different nanomaterials

Material/Synthesis method	Method	Linear range	LOD	Ref
CQD (Orange Juice)/Plasma	Fluorescence	1–14 μg mL^−1^	0.12 μg mL^−1^	This work
Boron and nitrogen Co-doped CQDs (citric acid and 2-amino-3-hydroxypyridine)/Hydrothermal	Fluorescence	2.5–22.5 mM	0.45 mM	44
CDs-Cu nanoclusters/Hydrothermal	Colorimetric detection	0–20 μM	0.6 μM	45
CQDs (Coconut Coir)/Calcination	Turn-on fluorescence	Not reported (NR)	0.00018 μg mL^−1^	46
Whole-cell biosensor	Fluorescence	0–200 nM	3 nM	47
Oxycarbide/Nafion electrode	Electrochemical	0–50 μg L^−1^ (0–0.45 μM)	3.97 ppb (35 nM)	48
N-CQDs/Hydrothermal	Fluorescence	20–300 μg L^−1^	20.69 μg L^−1^	49

The comparison reveals that the CQD sensor offers a competitive linear range and LOD, outperforming detection methods such as N-CQDs (LOD: 20.69 μg L^−1^) and CDs-Cu nanoclusters (LOD: 0.6 μM). However, its sensitivity is slightly lower than biosensor-based and electrochemical methods, such as the whole-cell biosensor (LOD: 3 nM) and oxycarbide/Nafion electrode (LOD: 3.97 ppb), which achieve ultra-low detection limits but require complex setups and expensive instrumentation. The CQD sensor stands out due to its green synthesis, using renewable *Citrus*-derived precursors, and its a cost-effectiveness compared to traditional Cd^2+^ detection methods involving toxic precursors or energy–intensive processes. While it may not match the detection sensitivity of advanced electrochemical sensors, it offers a balance of performance, simplicity, and sustainability, making it particularly attractive for environmental monitoring applications. In addition, the emissions of CQDs, prominently situated within the visible spectrum, render them highly advantageous for diverse practical applications in optoelectronic, bioimaging, and biomedical. Such versatility underscores the immense potential of CQDs as multifunctional nanomaterials in advancing various technological and biomedical endeavors.

Additionally, time-resolved PL measurement were conducted for the CQDs sample and CQDs + Cd^2+^, and the decay characteristic are shown in Fig. 7(c). The green curve plot represents the optimal fitting of the time decay curve using a three-exponential decay model described by eqn (3) as.^50^3
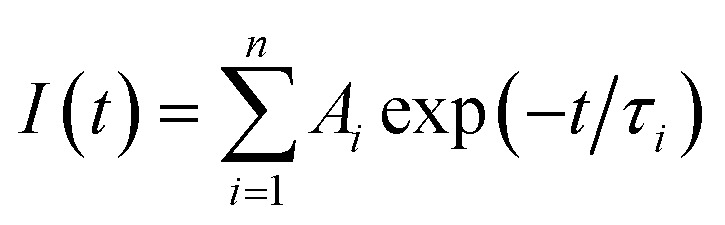
where *I* is the fluorescence intensity, *τ*_*i*_ denotes the decay components; and *A*_*i*_ represents the corresponding fitting constants. The average lifetime (*τ*_avg_) is estimated using the following eqn (4).4
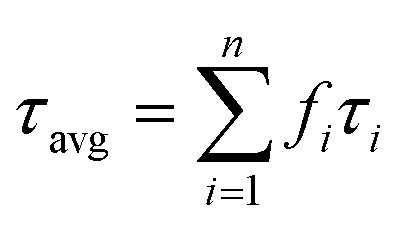
where *f*_*i*_ is the fractional contribution of each decay time to the steady-state intensity, which is given by eqn (4)5
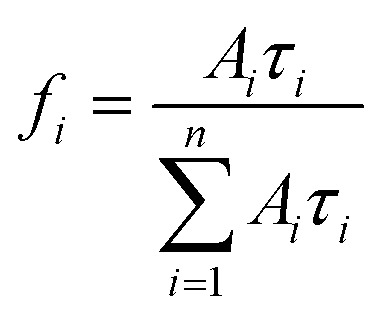


The fitting quality was assessed through the coefficient of determination (*R*^2^) and the dispersion of the weighted residuals (*n* = 3). The decay constants (*τ*_1_, *τ*_2_, and *τ*_3_) derived from the time-resolved photoluminescence (TRPL) measurements are summarized in Table 3. The average photoluminescence lifetime (*τ*_avg_) of the pristine CQDs was calculated to be 5.91 ns, which aligns well with previously reported values for CQDs synthesized *via* green or plasma-based approaches^18,23^ and is notably longer than lifetimes typically observed for CQDs synthesized *via* conventional pyrolysis or hydrothermal routes.^51^ This extended lifetime suggests a lower rate of non-radiative decay and a relatively lower density of surface defects, both of which favor efficient radiative recombination of charge carriers. In contrast, shorter PL lifetimes in other CQDs are generally associated with abundant surface trap states.^52,53^ The nanosecond-scale lifetime of our CQDs further supports their potential as stable and efficient fluorescent nanoprobes for applications in optoelectronics, biosensing, and bioimaging.^54^

**Table 3:** Parameters associated with decay characteristic of CQDs^a^

Samples	*τ*_1_	*τ*_2_	*τ*_3_	*τ*_avg_
CQDs	0.884	3.894	19.64	5.91
Cd^2+^ + CQDs	0.667	3.311	10.12	4.79

a Selectivity of CQDs for Cd^2+^ detection.

Upon the introduction of Cd^2+^ ions, the TRPL profile of the CQDs showed a multiexponential decay behavior, with a reduced average lifetime of 4.79 ns. All three decay components (*τ*_1_, *τ*_2_, and *τ*_3_) were shortened in the presence of Cd^2+^, indicating the emergence of additional non-radiative recombination pathways or the formation of new surface trap states upon metal ion coordination. These observations imply that Cd^2+^ ions interact with the surface functional groups of CQDs in a way that alters their surface electronic environment, promoting non-radiative decay and thereby contributing to the fluorescence quenching mechanism. This dynamic modulation of lifetime behavior provides strong evidence for Cd^2+^ induced photophysical changes, confirming the mechanistic basis for the “turn-off” fluorescence response in the sensing system.

To ensure the specificity of the CQDs for Cd^2+^ detection, their fluorescence response was evaluated in the presence of potentially interfering metal ions, including Na^+^, Cu^2+^, Mg^2+^, Ca^2+^, and Zn^2+^. As shown in Fig. 7(d), a significant fluorescence quenching effect was observed only in the presence of Cd^2+^, while negligible changes were recorded with other metal ions, confirming the high selectivity of the CQD-based sensor. The high specificity of CQDs for Cd^2+^ detection is attributed to strong coordination interactions between Cd^2+^ and the functional groups (–OH, –COOH) present on the CQDs surface, which facilitate non-radiative recombination pathways and enhance electron transfer efficiency. This selective binding mechanism ensures minimal interference from other metal ions, making CQDs a reliable and practical tool for environmental Cd^2+^ monitoring.

The CQD-based sensor presents a highly sensitive and selective fluorescence platform for Cd^2+^ detection, combining eco-friendly synthesis, cost-effectiveness, and competitive sensing performance. Its rapid fluorescence quenching response, strong Cd^2+^ affinity, and interference resistance highlight its potential for real-world applications in environmental monitoring and water safety assessments. Future research should focus on enhancing detection sensitivity through heteroatom doping (*e.g.*, N, S, B) or metal-functionalized CQDs, which can improve electron transfer efficiency and selectivity for Cd^2+^ ions. Additionally, expanding the detection scope to include real-world water samples with complex matrices will ensure the sensor's practical applicability in diverse environmental conditions. Another key advancement lies in developing portable sensing devices by integrating CQDs into miniaturized, field-deployable platforms for on-site Cd^2+^ monitoring, enabling rapid and cost-effective analysis.

## 4 Conclusion

This study successfully fabricated CQDs from *Citrus nobilis deliciosa* using a microplasma-assisted method, offering an eco-friendly, cost-effective, and scalable approach to producing highly functional nanomaterials. The resulting CQDs, demonstrated excitation-dependent fluorescence, high photostability and broad-spectrum antibacterial activity, achieving 99.24% and 98.12% growth suppression of *Staphylococcus aureus* and *Escherichia coli*, respectively, at 50 μg mL^−1^. The antibacterial mechanism was attributed to membrane destabilization and oxidative stress induction, positioning CQDs as promising candidates for next-generation antimicrobial agents. In addition to their antibacterial properties, the CQDs exhibited exceptional fluorescent sensing capabilities for Cd^2+^ detection, with a linear response range of 1–14 μg mL^−1^, a detection limit of 0.12 μg mL^−1^, and a Stern–Volmer quenching constant (*K*_sv_) of 0.45 μg m^L−1^. The high selectivity and sensitivity of the CQDs for Cd^2+^, combined with their resistance to interference from other metal ions, make them promising candidates for environmental monitoring and heavy metal detection. The dual functionality of CQDs serving as both antibacterial agents and fluorescent sensors underscores their potential for biomedical, environmental, and sensing applications. Their green synthesis, derived from a renewable *Citrus*-based precursor, aligns with the growing need for sustainable nanomaterials. Future research should focus on enhancing sensitivity through heteroatom doping or surface functionalization, expanding real-world applications, and integrating CQDs into portable sensing platforms for on-site heavy metal detection. By balancing performance, sustainability, and cost-effectiveness, CQDs synthesized *via* microplasma technology represent a versatile, scalable, and impactful nanomaterial for emerging scientific and industrial applications.

## Author contributions

N. M. H., L. D. T., L. A. T.: writing – original draft, visualization, software, methodology, investigation. N. M. H., T. N., L. D. T., L. A. T.: writing – review & editing, validation, supervision, resources, project administration, formal analysis, data curation, conceptualization. L. X. H., T. N., L. A. T.: validation, resources, data curation. M. H. N., L. A. T.: supervision, project administration, formal analysis, data curation.

## Conflicts of interest

There are no conflicts to declare.

## Data Availability

All data generated or analyzed during this study, including figures and tables, are available within the published article.
